# Impact of community-led enhanced structured health education on youths' knowledge of alcohol's effects in Kiambu county, Kenya: a quasi-experimental study

**DOI:** 10.11604/pamj.2025.51.34.47071

**Published:** 2025-06-09

**Authors:** Antony Kimata Mukui, John Paul Oyore, Mary Muiruri Gitahi

**Affiliations:** 1 Department of Family Medicine, Community Health and Epidemiology, Kenyatta University, Nairobi, Kenya

**Keywords:** Adverse effects, knowledge, DiD, Cohen’s d

## Abstract

**Introduction:**

alcohol is the most widely consumed substance globally, contributing to 2.6 million deaths annually, including 320,000 involving youths. In Africa, alcohol is linked to high mortality rates and increased Disability-adjusted Life Years. In Kiambu, Kenya, youth alcohol consumption stands at 28.9%, surpassing the national average of 11.2%. This study explores the impact of community-led, enhanced, structured health education on youths' awareness of alcohol's effects.

**Methods:**

a quasi-experimental study was conducted in Gatundu South (intervention) and Kabete (control) sub-counties in Kiambu from May 2022 to May 2023. Fourteen Community Health Promoters delivered monthly one-hour sessions in Gatundu South, totaling 12 sessions, covering 12 topics. Quantitative datasets were collected using the WHO's Alcohol AUDIT and questionnaire, while qualitative datasets were gathered through focus group discussions (FGDs) and key informant interviews (KIIs) and analyzed thematically. Kenyatta University's ethics committee granted study approval. R software supported data analysis, including mean knowledge scores, Chi-Square, ordinal logistic regression, Difference-in-Difference analysis, and Cohen's d.

**Results:**

youth in the intervention group experienced a significant increase in “very high knowledge” from 57% to 92.6%, compared to a smaller rise in the control group from 26.1% to 39.5%. Post-study, Kabete had 2.6% of participants in the very low knowledge category, versus 0% in Gatundu. The intervention showed a significant impact (DiD: 1.8, Cohen's d: 1.161). FGDs and KIIs indicated limited awareness and access to screening services.

**Conclusion:**

the intervention improved knowledge of alcohol-related harm, encouraged healthier behaviors, and reduced adverse effects.

## Introduction

Globally, alcohol is the most widely consumed substance and the first to be tried. It causes nearly 2.6 million deaths each year, including 320,000 among youths [1]. This burden is exacerbated by the high prevalence of alcohol consumption, with 23.5% of youths aged 15-19 being current drinkers. Despite this, many countries lack structured strategies to educate young people about alcohol´s harmful effects [2]. There is also limited understanding of youths' level of knowledge regarding these adverse effects and whether structured health education interventions can improve their knowledge levels.

In the African context, alcohol consumption among youth aged 15 to 24 is one of the leading predictors of Disability-adjusted Life Years (DALYs) and mortality. The impact on Disability-adjusted Life Years (DALYs) is particularly pronounced among young males [3]. The alcohol consumption rate among youth aged 15 to 19 years is estimated to be over 25% [4].

In Kenya, youth are exposed to alcohol at a young age due to a lack of understanding about the consequences, the widespread availability of alcohol, peer pressure, insufficient educational programs, and limited life skills to resist the allure of alcohol [5]. Although the National Agency for the Campaign Against Drug Abuse (NACADA) recommends a structured, youth-friendly health education program, county governments continue to struggle with implementing this intervention. Alcohol consumption has impacted the youth demographic, with approximately 13% of individuals aged 15-24 reporting alcohol use [6].

Notably, alcohol consumption among Kenyan youth is more prevalent among males, with figures indicating that approximately 20% of young males engage in alcohol use. For example, a study conducted in Kamirithu sub-location, Limuru sub-county revealed that 45% of male youth were moderate alcohol users, and 16.6% were classified as abusers, figures significantly higher than those recorded among their female counterparts [7].

The Central region has one of the highest rates of lifetime alcohol consumption among people aged 15 to 24 [6]. Kiambu County stands out as one of the most affected regions in Central Kenya. Approximately 28.9% of the youth in Kiambu have already consumed alcohol [8]. This is comparable to the rate of heavy episodic drinking among 15 to 19-year-olds in Kenya, estimated at 55.1% [9]

Alcohol consumption causes many morbidities [10]. It ranks as the sixth leading cause of preventable disability, disease, and death [11]. Young people who drink also experience consequences such as changes in appetite, eczema, weight loss, sleep disturbances, and headaches [11]. Additionally, youth who consume alcohol are at a higher risk of injuries, non-communicable diseases, such as liver cirrhosis, diabetes, and hypertension, and irresponsible sexual behaviors that raise the risk of HIV. To lessen alcohol´s side effects, young people are advised to reduce their intake [12]. Reduced alcohol consumption is associated with wider socio-economic benefits [13].

Despite the adverse effects, knowledge and awareness of alcohol-related harm among youth remain low in most societies [14]. Young people should participate in designing and implementing preventive health education programs [10]. Community-based education programs, like “Youth United Against Alcohol” by Blues Cross Kenya, demonstrate the importance of involving young people in alcohol prevention initiatives [15].

An evidence-based prevention intervention (Keepin' It Real) in Kenyan primary schools assessed the effectiveness of health education. It aimed to provide universal substance use prevention education tailored to the Kenyan context. Results indicated that the intervention was effective in increasing awareness about substance use risks [16]. Chaplin *et al*. [17] found that targeted educational interventions, including peer-to-peer education, can effectively enhance knowledge about health risks. Targeted interventions accelerate knowledge enhancement [18]. However, passive educational approaches often produce limited impacts on behavioral and cognitive outcomes [19]. According to Dodd *et al*. [20], continuous educational efforts significantly address knowledge gaps among youths. This is further supported by Manthey *et al*. [21], who found that interventions targeting both the health and social consequences of alcohol use resulted in improved knowledge retention and behavior change. This study is crucial in defining the most effective health education approach to combat alcoholism. It assesses the lasting effects of health education on the youth's understanding of adverse effects.

## Methods

**Study design:** the study was a quasi-experimental interventional type, employing a mixed-methods approach for collecting quantitative and qualitative data. It consisted of three phases: pre-intervention, intervention, and post-intervention. The pre-intervention and post-intervention surveys provided baseline and endline data on participants' knowledge regarding the effects of alcohol for both groups, respectively. The intervention phase involved administering health education sessions to the intervention group.

**Setting:** Kiambu County was chosen due to its high prevalence of alcohol consumption, particularly among the youth. Kiambu is county number 22 in Kenya and features high-elevation plains, plateaus, and hills. The area is situated between 1,500 and 1,800 meters above sea level, with primary activities including dairy farming and tea cultivation, while residents also engage in maize farming and horticulture. The two sub-counties selected for study (Gatundu South and Kabete) are far apart to minimize contamination. In each sub-county, three wards were randomly selected: Gatundu South (Ngenda, Kiganjo, and Kiamwangi wards) and Kabete (Kabete, Nyathuna, and Muguga wards).

Recruitment and the initial health education session took place in May 2022, lasting one year and concluding in May 2023, with each session lasting one hour. A total of 26 Community Health Promoters (CHPs) were selected and trained before the study. For the intervention group, 14 CHPs conducted a baseline survey, implemented the enhanced structured health education, and carried out an endline survey. For the control group, 12 CHPs performed a baseline survey, provided routine health education, and conducted an endline survey. Five recovered alcoholics also supported the CHPs by giving motivational talks. Data collection involved screening and categorizing youth who consume alcohol into four risk categories (low, risky-hazardous, harmful, and high risk) using the WHO Alcohol Use Disorders Identification Test (AUDIT) tool. The level of knowledge of each recruited youth regarding the adverse effects of alcohol was assessed using a questionnaire.

The study included 12 Focus Group Discussions (FGDs), each with six participants, totaling 72 individuals, along with 20 Key Informant Interviews (KIIs), all conducted by three Community Health Assistants in each subcounty. In every subcounty, one FGD was held per ward at baseline and another at endline. Random sampling was used to select the specific Community Health Promoter (CHP) and the subsequent six participants associated with the chosen CHP for the FGD. For the KIIs, purposive sampling was employed to identify 20 participants from key community leaders, including the County CHP coordinator, subcounty coordinators, Community Health Assistants, chiefs, Public Health Officers, youth leaders, and motorbike riders.

Twelve topics were addressed, including understanding alcohol: myths, facts, and realities; risk levels and patterns of alcohol use; alcohol and youth: why it´s riskier at this age; sociodemographic factors and alcohol use; health effects of alcohol use; non-health effects of alcohol use; legal and social consequences of underage drinking; alcohol, mental health, and emotional well-being; building life skills for resisting alcohol use; alternatives to alcohol: healthy lifestyles for youth; and the role of family, peers, and community in alcohol prevention and management, along with structured health education and behavior change. Boarding students participated in the sessions during the midterm and end-of-term holidays. The sessions emphasized prevention strategies, life skills, social skills, self-management, and information concerning alcohol use.

**Figure 1:**
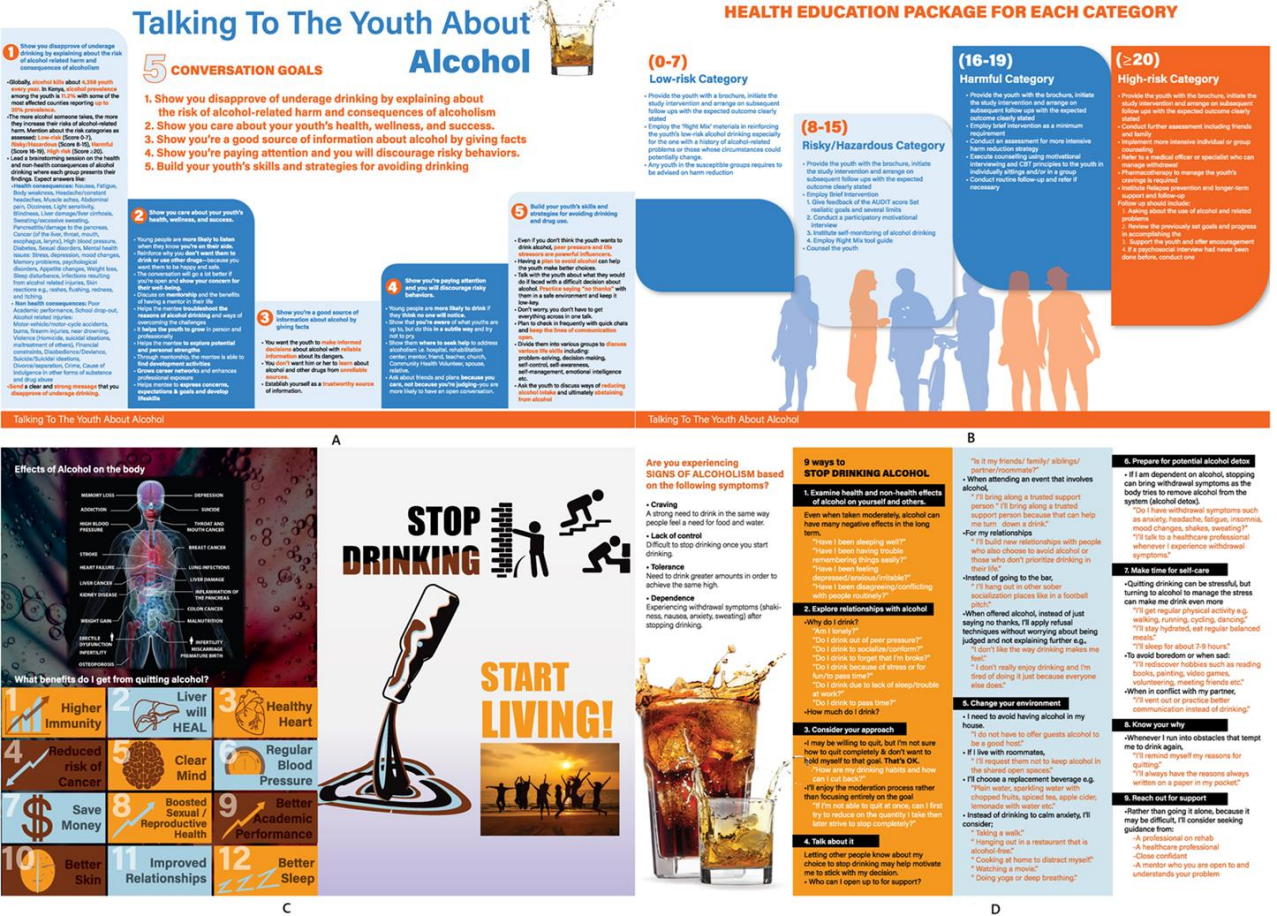
community health promoter and youth flyer

Every youth received a flyer to read regularly at home and collectively during the sessions. The flyer provided information on the signs of alcoholism, methods for reducing alcohol consumption, the effects of alcohol, and the benefits of quitting. Flyers for the youth and community health promoters were designed as shown in Figure 1. As illustrated in Figure 1, the effects are detailed in a diagram of human anatomy highlighting areas affected by alcohol, designed to instill fear, according to the Extended Parallel Process Model (EPM). This model fosters positive change by inducing fear of unhealthy behaviors. It predicts how individuals respond when faced with fear-inducing stimuli and promotes the adoption of healthy behaviors [22]. The model attributes the intervention's outcome to four predictive factors: self-efficacy, susceptibility, response efficacy, and severity. Perceived severity and susceptibility are considered threat variables. Severity refers to the seriousness of the consequences of a certain behavior, while susceptibility relates to the likelihood of those consequences occurring. Conversely, response efficacy and self-efficacy are classified as efficacy variables, with response efficacy reflecting the effectiveness of the proposed solution, whereas self-efficacy pertains to an individual's confidence in their ability to successfully implement the proposed solution.

**Participants:** alcohol-drinking youth aged 15 to 24 who consented to participate were selected. Minors sought permission from their parents or guardians. The study involved the 26 registered Community Health Promoters (CHPs) in the area and five recovered alcoholics who agreed to participate. The lead subcounty coordinator in each subcounty recruited the CHPs. Each CHP had to be registered, actively working in the selected ward, willing to participate in the study, and deemed youth-friendly by the lead. Before youth recruitment, each recruited CHP received training.

**Variables:** the independent variables in this study encompassed sociodemographic factors, which were measured pre- and post-study. The dependent variable was the knowledge level of alcohol-related harm, evaluated pre- and post-study. Some potential confounding variables included previous exposure to alcohol education programs, religious and cultural beliefs, and family history of alcohol use.

**Data sources/measurement:** during the baseline survey, participants completed a researcher-administered questionnaire to capture their sociodemographic characteristics (gender, age, ward, settlement, level of education, employment status, those living with parents/relatives, money received from the parent in a month, and marital status) and their knowledge (dependent variable) regarding alcohol-related harm. For knowledge assessment, there were 38 multiple-choice questions to choose from. Based on the identification of correct effects of alcohol among the 38 provided options, participants´ knowledge scores were categorized as follows: very low knowledge (0%-20%), low knowledge (21%-40%), moderate knowledge (41%-60%), high knowledge (61%-80%), or very high knowledge (81%-100%). The five quantiles were adopted from Sitaula *et al*. [23]. The reliability of the questionnaire was evaluated using Cronbach´s alpha test before the start of the study.

**Bias:** the study employed standardized approaches to minimize potential sources of bias by training the CHPs before the research and allowing them to facilitate the recruitment process independently. The WHO alcohol AUDIT tool was used for screening to reduce information bias [24]. To minimize selection bias, participants were randomly selected but were chosen according to standard inclusion and exclusion criteria. The two sub-counties are also sufficiently distant to minimize contamination.

**Study size:** in Gatundu, CHPs recruited 156 youth in Kiganjo, 132 in Ngenda, and 68 in Kiamwangi. In Kabete, screening yielded 145 in Kabete, 109 in Nyathuna, and 102 in Muguga. The three Gatundu wards (Kiganjo, Ngenda, and Kiamwangi) encompass 29,146 households, with approximately 8,744 households containing alcohol-drinking youth (30% of 29,146). Kabete's three wards (Kabete, Nyathuna, and Muguga) consist of 23,099 households, with about 6,930 households having youth who drink alcohol. The sample size calculation was performed using a formula aligned with the quasi-experimental design employed by Charan and Biswas [25].


n=Zα22P1−P+ZβP11−P1+P21−P2P2−P12

Where, n= desired sample size, α= type I error (0.05), β= type II error (0.10). At 95% confidence, Z_α/2_)= 1.96. At 80% power, Z_β_= 0.842. P= pooled proportion (the average of P_1_ and P_2_). P_1_= 55% estimated proportion at risk of alcohol-related harm pre-study (borrowed from the prevalence of heavy episodic drinking among the youth estimated at 55%). P_2_= 44% estimated proportion at risk of alcohol related harm post-study


$$P_{1}-P_{2}=Effect\;size\left(10\%\right):\left(0.44-0.55\right)=-0.11,P=\frac{P_{1}+P_{2}}{2}=\frac{0.55+0.44}{2}=0.495$$

$$n={\left[\frac{1.96\sqrt{2*0.495\left(1-0.495\right)}+0.842\sqrt{0.55\left(1-0.55\right)+0.44\left(1-0.44\right)}}{\left(0.44-0.55\right)}\right]}^{2}=324$$

Thus, the minimum required sample size was estimated at 324. The sample was then adjusted by 10% to allow for attrition; hence, (324 + (324 * 0.10)) = 356. Therefore, the final sample size was raised to 356 respondents. There were 356 participants in the intervention group and 356 in the control group, totaling 712 participants.

**Inclusion and exclusion criteria:** alcohol-drinking youth aged 15-24 years in the selected communities who were willing to give consent were recruited. Minors sought guardian or parental consent. Finally, the lead county coordinator selected the CHPs willing to participate.

**Quantitative and qualitative variables:** the questionnaire helped to measure knowledge and sociodemographic variables, while FGD and KII provided qualitative data.

**Statistical methods:** R software was utilized to analyze descriptive and inferential statistics. Tables, charts, and figures illustrate the quantitative data, while qualitative data was presented in thematic order. Chi-Square was employed to determine the association between variables. The use of ordinal logistic regression helped establish the predictors of youths' knowledge levels, while Difference-in-Difference (DiD), Cohen´s d, and mean risk scores measured the intervention´s impact.

**Ethical consideration:** ethical approval was granted by the Graduate School of Kenyatta University and the Kenyatta University Ethics Review Committee, with approval number PKU/2473/11601. Permission was also obtained from NACOSTI (the National Commission for Science, Technology, and Innovation) and the Kiambu County government. The CHPs and coordinators assisted with community engagement. Participants signed an informed consent form, while guardians or parents provided consent for minors. Data collection tools were coded and securely stored. Controls continued to receive health education as it had been traditionally offered.

## Results

**Participants:** the study recruited a total of 712 participants, with 356 assigned to each of the intervention and control arms, as shown in Table 1. By the end of the year, four participants dropped out of the intervention arm and seven from the control arm, resulting in 352 participants in the intervention group and 349 in the control group.

**Table 1:** distribution of the participants' demographic characteristics

	Intervention		Control	
	Pre-study	Post-study	Pre-study	Post-study
	N = 356	N = 352	N = 356	N = 349
**Gender**				
Male	280 (79%)	276 (78%)	251 (71%)	248 (71%)
Female	76 (21%)	76 (22%)	105 (29%)	101 (29%)
**Age**				
15-19	128 (36%)	127 (36%)	105 (29%)	103 (30%)
20-24	228 (64%)	225 (64%)	251 (71%)	246 (70%)
**Education level**				
Uneducated	11 (3.1%)	11 (3.1%)	5 (1.4%)	5 (1.4%)
Primary	65 (18%)	64 (18%)	0 (0%)	58 (17%)
Secondary	166 (47%)	166 (47%)	234 (66%)	171 (49%)
Polytechnic	15 (4.2%)	15 (4.3%)	23 (6.5%)	23 (6.6%)
College	75 (21%)	73 (21%)	73 (21%)	70 (20%)
University	24 (6.7%)	23 (6.5%)	21 (5.9%)	22 (6.3%)
**Marital status**				
Married	24 (6.7%)	24 (6.8%)	24 (6.7%)	24 (6.9%)
Single	317 (89%)	313 (89%)	314 (88%)	308 (88%)
Divorced	3 (0.8%)	3 (0.9%)	6 (1.7%)	5 (1.4%)
Separated	10 (2.8%)	10 (2.8%)	11 (3.1%)	11 (3.2%)
Widowed	2 (0.6%)	2 (0.6%)	1 (0.3%)	1 (0.3%)
**Settlement**				
Rural	335 (94%)	331 (94%)	322 (90%)	315 (90%)
Suburban	21 (5.9%)	21 (6.0%)	34 (9.6%)	34 (9.7%)
**Employment status**				
Unemployed	318 (89%)	302 (86%)	238 (67%)	232 (66%)
Employed	38 (11%)	50 (14%)	118 (33%)	117 4%)
**Live with a parent/guardian**				
Yes	292(82%)	287(81.5%)	245(69%)	241(69%)
No	64(18%)	65(18.5%)	111(31%)	108(31%)

**Descriptive data:** the population, predominantly male and aged 20-24, presented a demographic picture of alcohol-consuming youth in Kiambu county, with the demographic pattern in the pre-study resembling the post-study findings (Table 1). In the pre-study, the intervention group consisted of 79% males, while the control group comprised 71% males. The majority, 64% (intervention group) and 71% (controls), were aged 20-24 years. Most participants had completed at least secondary education, with 47% (intervention) and 66% (control) having done so. Most participants were single, with 89% (intervention) and 88% (control). Additionally, the majority lived in rural areas, with 94% (intervention) and 90% (control). Unemployment rates were high, with 89% in the intervention group and 67% in the control group. Regarding living arrangements, most participants resided with their parents or guardians, comprising 82% (intervention) and 69% (control).

**Outcome data:** the primary study outcome was knowledge regarding the effects of alcohol. A questionnaire was designed to assess knowledge and measure changes; the results are discussed below.

**Figure 2:**
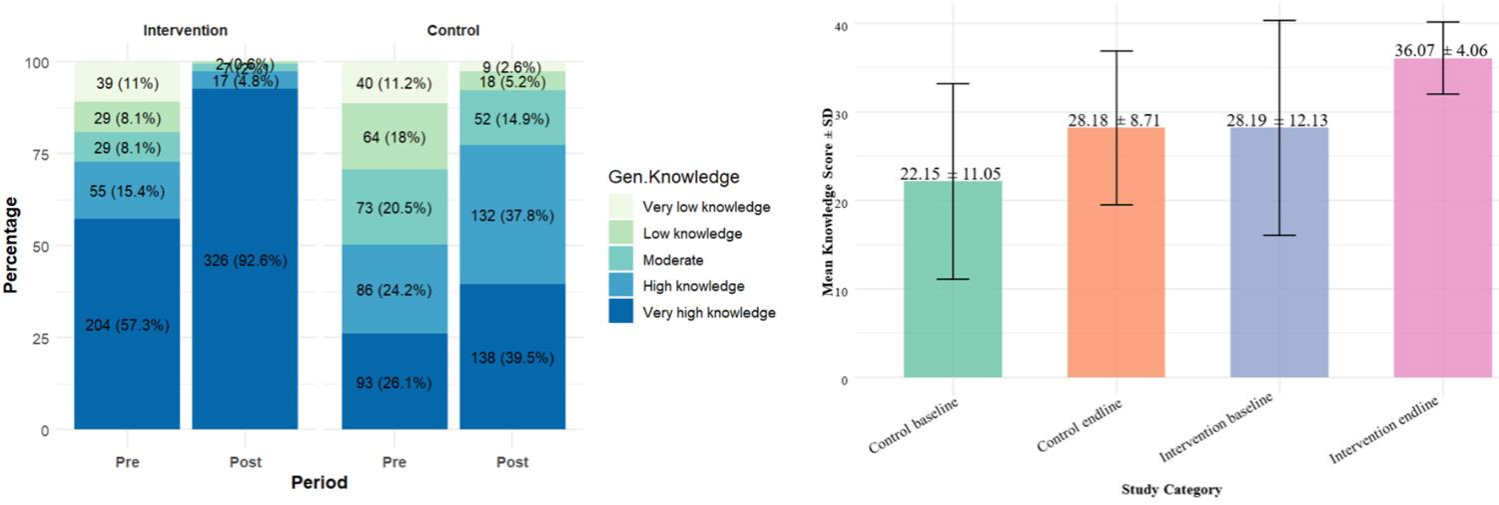
knowledge level and knowledge scores

**Main results:** in a graphical representation of the change in knowledge about the adverse effects of alcohol among youth, as shown in the stacked bar chart in Figure 2, knowledge increased in both groups. However, a very high level of knowledge is the most desirable to motivate behavior change. This level was primarily achieved in the intervention group. In the stacked bar chart in Figure 2, the percentage of the intervention group that ranked in the very high knowledge category rose from 57% pre-study to 92.6% post-study. In comparison, the control group for the same knowledge category exhibited a modest increase from 26.1% to 39.5%.

Furthermore, post-study, 2.6% of the control group participants fell into the very low knowledge category, compared to 0% in the intervention group. Furthermore, the results of mean knowledge scores in the bar graph in Figure 2 indicate that both groups experienced improvements in their mean knowledge scores; however, the intervention group showed a more significant increase, with its mean score rising from 28.19 ± 12.13 to 36.07 ± 4.06. This suggests that the participants' mean scores shifted from a high to a very high knowledge category. While the control group experienced a slight increase from a mean of 22.15 ± 11.05 (moderate) to 28.18 ± 8.71 (high), the improvement may be confounded by prior exposure to the knowledge questions during the baseline assessment.

In terms of the association between knowledge and sociodemographic characteristics for the intervention group at pre-study, as shown in Table 2, age, education level, settlement type, whether the youth lived with their parents or relatives, and employment status exhibited statistically significant associations with knowledge level (p<0.05). The most knowledgeable participants were those aged 20-24, polytechnic students, rural residents, individuals living with their parents, and those who were employed. However, this association was not evident after the study. For the control group, at the pre-study stage, education level and whether the youth lived with their parents were statistically significant (p<0.05). The most knowledgeable individuals were college students and those living with their parents (Table 3). At the post-study stage, age, settlement type, and the amount spent on alcohol each month were statistically significant, with the most knowledgeable individuals being 15-19-year-olds, rural residents, and those spending less than KES 10,000 monthly on alcohol.

**Table 2:** association between level of knowledge and sociodemographic characteristics in the intervention group

Intervention												
Characteristic	Pre-study						Post-study					
	Very low knowledge	Low knowledge	Moderate	High knowledge	Very high knowledge	p-valuee^2^	Very low knowledge	Low knowledge	Moderate	High knowledge	Very high knowledge	p-value^2^
	N = 39^1^	N = 29^1^	N = 29^1^	N = 55^1^	N = 204^1^		N = 0^1^	N = 2^1^	N = 7^1^	N = 17^1^	N = 326^1^	
**Gender**						0.2						0.3
Male	33 (12%)	24 (8.6%)	24 (8.6%)	37 (13%)	162 (58%)		0 (0%)	1 (0.4%)	4 (1.4%)	15 (5.4%)	256 (93%)	
Female	6 (7.9%)	5 (6.6%)	5 (6.6%)	18 (24%)	42 (55%)		0 (0%)	1 (1.3%)	3 (3.9%)	2 (2.6%)	70 (92%)	
**Age**						0.008						0.7
15-19	22 (17%)	13 (10%)	10(7.8%)	24 (19%)	59 (46%)		0 (0%)	1 (0.8%)	1 (0.8%)	6 (4.7%)	119 (94%)	
20-24	17 (7.5%)	16 (7.0%)	19(8.3%)	31 (14%)	145 (64%)		0 (0%)	1 (0.4%)	6 (2.7%)	11 (4.9%)	207 (92%)	
**Level of education**						<0.001						>0.9
Uneducated	0 (0%)	0 (0%)	3 (27%)	0 (0%)	8 (73%)		0 (0%)	0 (0%)	0 (0%)	0 (0%)	11 (100%)	
Primary	9 (14%)	5 (7.7%)	3 (4.6%)	14 (22%)	34 (52%)		0 (0%)	0 (0%)	2 (3.1%)	5 (7.8%)	57 (89%)	
Secondary	18 (11%)	9 (5.4%)	13(7.8%)	32 (19%)	94 (57%)		0 (0%)	1 (0.6%)	2 (1.2%)	8 (4.8%)	155 (93%)	
Polytechnic	0 (0%)	0 (0%)	1 (6.7%)	1 (6.7%)	13 (87%)		0 (0%)	0 (0%)	0 (0%)	1 (6.7%)	14 (93%)	
College	12 (16%)	14 (19%)	8 (11%)	6 (8.0%)	35 (47%)		0 (0%)	1 (1.4%)	2 (2.7%)	3 (4.1%)	67 (92%)	
University	0 (0%)	1 (4.2%)	1 (4.2%)	2 (8.3%)	20 (83%)		0 (0%)	0 (0%)	1 (4.3%)	0 (0%)	22 (96%)	
**Settlement**						0.039						0.6
Rural	39 (12%)	29 (8.7%)	25(7.5%)	49 (15%)	193 (58%)		0 (0%)	2 (0.6%)	7 (2.1%)	17 (5.1%)	305 (92%)	
Suburban	0 (0%)	0 (0%)	4 (19%)	6 (29%)	11 (52%)		0 (0%)	0 (0%)	0 (0%)	0 (0%)	21 (100%)	
**Marital status**						0.3						0.9
Married	0 (0%)	0 (0%)	5 (21%)	4 (17%)	15 (63%)		0 (0%)	0 (0%)	0 (0%)	0 (0%)	24 (100%)	
Single	39 (12%)	27 (8.5%)	23(7.3%)	48 (15%)	180 (57%)		0 (0%)	2 (0.6%)	6 (1.9%)	17 (5.4%)	288 (92%)	
Divorced	0 (0%)	0 (0%)	0 (0%)	0 (0%)	3 (100%)		0 (0%)	0 (0%)	0 (0%)	0 (0%)	3 (100%)	
Separated	0 (0%)	2 (20%)	1 (10%)	2 (20%)	5 (50%)		0 (0%)	0 (0%)	1 (10%)	0 (0%)	9 (90%)	
Widowed	0 (0%)	0 (0%)	0 (0%)	1 (50%)	1 (50%)		0 (0%)	0 (0%)	0 (0%)	0 (0%)	2 (100%)	
**Live with parents/relatives**						0.05						0.7
Yes	34 (12%)	21 (7.2%)	19(6.5%)	44 (15%)	174 (60%)		0 (0%)	2 (0.7%)	5 (1.7%)	13 (4.5%)	267 (93%)	
No	5 (7.8%)	8 (13%)	10 (16%)	11 (17%)	30 (47%)		0 (0%)	0 (0%)	2 (3.1%)	4 (6.2%)	59 (91%)	
**Employment Status**						0.035						0.15
Unemployed	39 (12%)	29 (9.1%)	26(8.2%)	48 (15%)	176 (55%)		0 (0%)	2 (0.7%)	4 (1.3%)	14 (4.6%)	282 (93%)	
Employed	0 (0%)	0 (0%)	3 (7.9%)	7 (18%)	28 (74%)		0 (0%)	0 (0%)	3 (6.0%)	3 (6.0%)	44 (88%)	
**Money spent on alcohol per month**						0.2						0.5
Zero spend	0 (NA%)	0 (NA%)	0 (NA%)	0 (NA%)	0 (NA%)		0 (0%)	1 (0.9%)	3 (2.7%)	3 (2.7%)	103 (94%)	
1-10,000	39 (11%)	29 (8.4%)	26(7.5%)	55 (16%)	196 (57%)		0 (0%)	1 (0.4%)	4 (1.7%)	14 (5.8%)	223 (92%)	
10,001-20000	0 (0%)	0 (0%)	3 (30%)	0 (0%)	7 (70%)		0 (NA%)	0 (NA%)	0 (NA%)	0 (NA%)	0 (NA%)	
20,001+	0 (0%)	0 (0%)	0 (0%)	0 (0%)	1 (100%)		0 (NA%)	0 (NA%)	0 (NA%)	0 (NA%)	0 (NA%)	

1 n (%) Pearson’s Chi-squared test

**Table 3:** association between level of knowledge and sociodemographic characteristics in the control group

Control												
Characteristic	Pre-study						Post-study					
	Very low knowledge	Low knowledge	Moderate	High knowledge	Very high knowledge	p-value^2^	Very low knowledge	Low knowledge	Moderate	High knowledge	Very high knowledge	p-value^2^
	N = 40^1^	N = 64^1^	N = 73^1^	N = 86^1^	N = 93^1^		N = 9^1^	N = 18^1^	N = 52^1^	N = 132^1^	N = 138^1^	
**Gender**						0.06						0.3
Male	32 (13%)	43 (17%)	58 (23%)	52 (21%)	66 (26%)		9 (3.6%)	14 (5.6%)	39 (16%)	93 (38%)	93 (38%)	
Female	8 (7.6%)	21 (20%)	15 (14%)	34 (32%)	27 (26%)		0 (0%)	4 (4.0%)	13 (13%)	39 (39%)	45 (45%)	
**Age**						0.8						0.043
15-19	11 (10%)	20 (19%)	25 (24%)	24 (23%)	25 (24%)		2 (1.9%)	3 (2.9%)	15 (15%)	30 (29%)	53 (51%)	
20-24	29 (12%)	44 (18%)	48 (19%)	62 (25%)	68 (27%)		7 (2.8%)	15 (6.1%)	37 (15%)	102 (41%)	85 (35%)	
**Level of education**						<0.001						0.4
Uneducated	0 (0%)	4 (80%)	0 (0%)	1 (20%)	0 (0%)		0 (0%)	0 (0%)	0 (0%)	2 (40%)	3 (60%)	
Primary	0 (NA%)	0 (NA%)	0 (NA%)	0 (NA%)	0 (NA%)		4 (6.9%)	1 (1.7%)	9 (16%)	28 (48%)	16 (28%)	
Secondary	37 (16%)	40 (17%)	51 (22%)	51 (22%)	55 (24%)		3 (1.8%)	9 (5.3%)	28 (16%)	61 (36%)	70 (41%)	
Polytechnic	0 (0%)	7 (30%)	7 (30%)	5 (22%)	4 (17%)		1 (4.3%)	2 (8.7%)	2 (8.7%)	5 (22%)	13 (57%)	
College	2 (2.7%)	11 (15%)	10 (14%)	23 (32%)	27 (37%)		0 (0%)	5 (7.1%)	9 (13%)	29 (41%)	27 (39%)	
University	1 (4.8%)	2 (9.5%)	5 (24%)	6 (29%)	7 (33%)		1 (4.5%)	1 (4.5%)	4 (18%)	7 (32%)	9 (41%)	
**Settlement**						0.2						0.033
Rural	36 (11%)	54 (17%)	66 (20%)	83 (26%)	83 (26%)		9 (2.9%)	17 (5.4%)	47 (15%)	111 (35%)	131 (42%)	
Suburban	4 (12%)	10 (29%)	7 (21%)	3 (8.8%)	10 (29%)		0 (0%)	1 (2.9%)	5 (15%)	21 (62%)	7 (21%)	
**Marital status**						0.11						0.058
Married	2 (8.3%)	6 (25%)	5 (21%)	6 (25%)	5 (21%)		0 (0%)	1 (4.2%)	4 (17%)	10 (42%)	9 (38%)	
Single	37 (12%)	49 (16%)	63 (20%)	78 (25%)	87 (28%)		9 (2.9%)	15 (4.9%)	45 (15%)	114 (37%)	125 (41%)	
Divorced	0 (0%)	3 (50%)	2 (33%)	1 (17%)	0 (0%)		0 (0%)	0 (0%)	0 (0%)	4 (80%)	1 (20%)	
Separated	1 (9.1%)	6 (55%)	2 (18%)	1 (9.1%)	1 (9.1%)		0 (0%)	1 (9.1%)	3 (27%)	4 (36%)	3 (27%)	
Widowed	0 (0%)	0 (0%)	1 (100%)	0 (0%)	0 (0%)		0 (0%)	1 (100%)	0 (0%)	0 (0%)	0 (0%)	
**Live with parents/relatives**						0.052						0.3
Yes	34 (14%)	41 (17%)	52 (21%)	62 (25%)	56 (23%)		4 (1.7%)	13 (5.4%)	38 (16%)	86 (36%)	100 (41%)	
No	6 (5.4%)	23 (21%)	21 (19%)	24 (22%)	37 (33%)		5 (4.6%)	5 (4.6%)	14 (13%)	46 (43%)	38 (35%)	
**Employment Status**						0.2						0.14
Unemployed	30 (13%)	37 (16%)	50 (21%)	63 (26%)	58 (24%)		7 (3.0%)	15 (6.5%)	29 (13%)	84 (36%)	97 (42%)	
Employed	10 (8.5%)	27 (23%)	23 (19%)	23 (19%)	35 (30%)		2 (1.7%)	3 (2.6%)	23 (20%)	48 (41%)	41 (35%)	
**Money spent on alcohol per month**						0.3						0.02
Zero spend	0 (0%)	1 (100%)	0 (0%)	0 (0%)	0 (0%)		0 (NA%)	0 (NA%)	0 (NA%)	0 (NA%)	0 (NA%)	
1-10,000	40 (12%)	61 (18%)	69 (20%)	83 (24%)	88 (26%)		7 (2.1%)	16 (4.8%)	49 (15%)	127 (38%)	134 (40%)	
10,001-20000	0 (0%)	2 (20%)	4 (40%)	2 (20%)	2 (20%)		2 (14%)	1 (7.1%)	3 (21%)	4 (29%)	4 (29%)	
20,001+	0 (0%)	0 (0%)	0 (0%)	1 (25%)	3 (75%)		0 (0%)	1 (50%)	0 (0%)	1 (50%)	0 (0%)	

1 n (%) Pearson’s Chi-squared test

Regarding predictors of knowledge pre-study, as demonstrated by ordinal logistic regression in Table 4, age, ward type, education level, and the amount of money received from parents were identified as significant factors. The categories with higher odds of knowledge included individuals aged 20-24 years (in the univariate analysis), Ngenda (in the univariate analysis), Kiganjo (in the multivariate analysis), those with a university education (in both the univariate and multivariate analyses), and those receiving a monthly income of KES 10,000-20,000 from their parents (in the univariate analysis). In the post-study phase, the main predictors of knowledge included age, type of ward, employment status, and whether the youth lived with their parents (Table 4). Those with higher odds of possessing knowledge were individuals aged 15-19 years (in the univariate analysis), residents of Kiamwangi (in the univariate analysis), the unemployed (in the univariate analysis), and individuals living with their parents (in the univariate analysis).

**Table 4:** univariate and multivariate ordinal logistic regression model for the pre- and post-study period

Characteristics	Pre-study							Post-study						
	Univariate				Multivariable			Univariate				Multivariable		
	N	OR^1^	95% CI^1^	p-value	OR^1^	95% CI^1^	p-value	N	OR^1^	95% CI^1^	p-value	OR1	95% CI^1^	p-value
**Gender**	712			0.78			0.52	701			0.83			0.88
Male									—	—		—	—	
Female		1.04	0.77, 1.41		0.84	0.50, 1.43			0.96	0.68, 1.37		1.06	0.53, 2.11	
**Age**	712			0.018			0.29	701			<0.001			0.18
15-19		—	—		—	—			—	—		—	—	
20-24		1.41	1.06, 1.87		0.75	0.44, 1.28			0.56	0.39, 0.79		0.63	0.32, 1.23	
**Ward**	712			<0.001			<0.001	701			<0.001			<0.001
Kiganjo		—	—		—	—			—	—		—	—	
Kiamwangi		1.1	0.61, 2.01		0.02	0.00, 0.05			1.07	0.34, 4.01		0		
Ngenda		2.42	1.53, 3.84		0.34	0.12, 0.89			0.66	0.27, 1.59		0		
Kabete		1	0.64, 1.56		0.05	0.02, 0.12			0.04	0.02, 0.09		0		
Muguga		0.2	0.12, 0.31		0.02	0.01, 0.06			0.04	0.02, 0.09		0		
Nyathuna		0.63	0.41, 0.98		0.06	0.02, 0.18			0.05	0.02, 0.10		0		
**Settlement**	712			0.58			0.66	701			0.06			0.41
Rural		—	—		—	—			—	—		—	—	
Suburban		0.87	0.54, 1.43		1.32	0.39, 4.87			0.61	0.37, 1.02		1.72	0.48, 6.96	
**Level of education**	712			0.012			<0.001	701			0.29			0.22
Uneducated		—	—		—	—			—	—		—	—	
Primary		1.21	0.42, 3.33		0.37	0.03, 3.18			0.21	0.03, 0.79		0.35	0.01, 4.62	
Secondary		0.7	0.26, 1.73		0.23	0.02, 1.51			0.27	0.04, 0.99		0.54	0.02, 6.56	
Polytechnic		0.99	0.33, 2.85		0.49	0.04, 3.81			0.32	0.05, 1.38		2.3	0.08, 40.4	
College		0.82	0.30, 2.10		1.52	0.15, 10.7			0.27	0.04, 0.99		0.84	0.03, 10.3	
University		1.83	0.61, 5.30		1.92	0.17, 15.9			0.29	0.04, 1.20		0.56	0.02, 8.40	
**Employment status**	712			0.96			0.18	701			<0.001			0.67
Unemployed		—	—		—	—			—	—		—	—	
Employed		0.99	0.72, 1.36		0.5	0.18, 1.39			0.46	0.33, 0.64		1.29	0.40, 4.42	
**Live with parents/relatives**	712			0.59			0.95	701			0.003			0.93
Yes		—	—		—	—			—	—		—	—	
No		0.92	0.68, 1.25		0.98	0.43, 2.22			0.59	0.42, 0.83		0.96	0.37, 2.55	
**Money received from the parents in a month**	284			<0.001			0.32	270			0.17			0.47
1-10,000		—	—		—	—			—	—		—	—	
10,001-20,000		6.29	2.52, 19.1		1.85	0.52, 7.45			2.93	0.97, 12.7		0.65	0.11, 4.64	
20,001-30,000		2.76	0.90, 10.3		3.15	0.79, 14.8			0.72	0.22, 2.78		0.53	0.10, 2.88	
30,001-40,000		1.78	0.43, 8.86		0.77	0.15, 4.62			0.4	0.06, 3.26		0.18	0.02, 2.01	
**Marital status**	712			0.33			0.054	701			0.42			0.27
Married		—	—		—	—			—	—		—	—	
Single		0.9	0.53, 1.50		7.74	1.20, 49.4			0.88	0.46, 1.61		0.53	0.02, 6.08	
Others		0.58	0.27, 1.25		16.7	1.52, 199			0.57	0.23, 1.40		0.07	0.00, 2.07	

1 OR = Odds Ratio, CI = confidence interval

**Figure 3:**
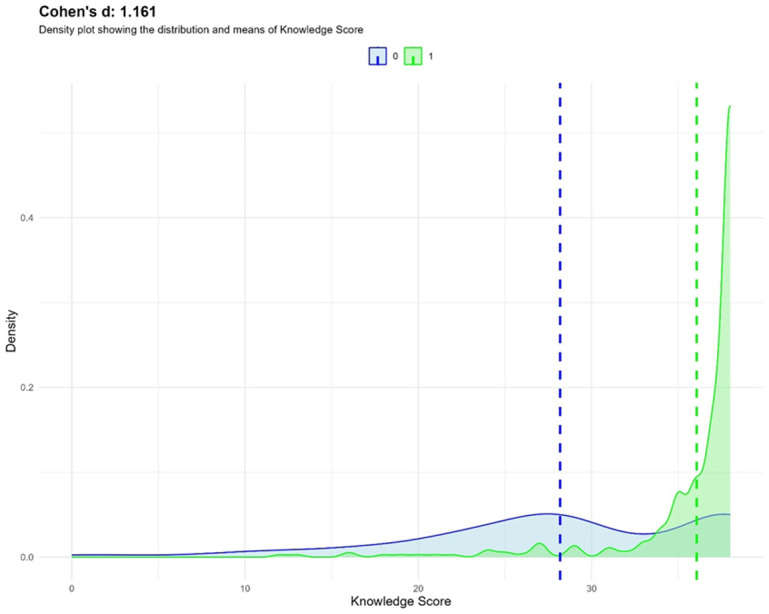
impact assessment using Cohen’s d; density plot showing the distribution and means of knowledge score

To further demonstrate the impact of the intervention, the bar graph in Figure 3 shows that the significant shift in mean knowledge scores in the intervention group compared to the control yielded a positive DiD value of 1.84. This indicates that the health education in the intervention group had a more substantial effect on increasing the knowledge of the youth. Moreover, the density plot in Figure 3 demonstrates a Cohen´s d of 1.161, indicating a large impact. The blue distribution for the control group (0) shows a relatively lower and broader range of knowledge scores. In contrast, the green distribution for the intervention group (1) exhibits a clear rightward shift, indicating significantly higher knowledge scores concentrated in the 20-30 range. The non-overlapping nature of these distributions and the clear separation of their means (represented by the dashed vertical lines) further emphasize the effectiveness of the intervention in improving participants' knowledge outcomes.

**Figure 4:**
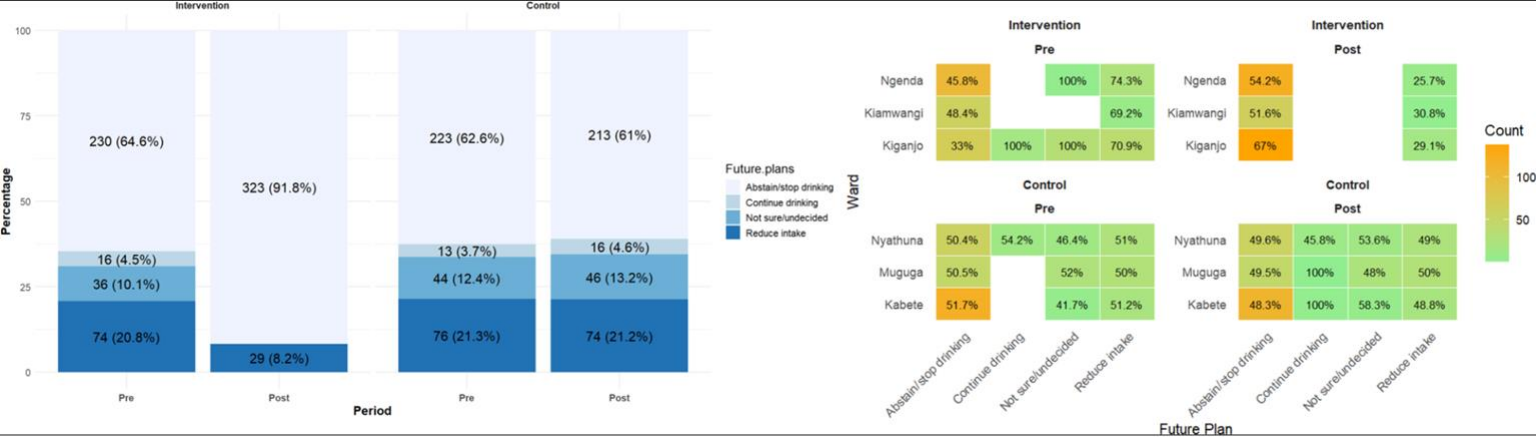
plan distribution per ward in the study group

**Other analyses:** the knowledge levels also appeared to affect future drinking plans, as illustrated in Figure 4. In the intervention group, 4.5% of youth initially indicated they would continue drinking, but after the health education sessions, none (0%) expressed a desire to continue. Furthermore, the percentage of those who stated they would abstain rose from 64.6% to 91.8%. In the control group, the proportion of those who intended to continue drinking increased from 3.7% pre-study to 4.6% post-study. Additionally, there was a decline in the percentage of youths who said they would abstain, dropping from 62.6% pre-study to 61% post-study. The youth in Kiganjo ward (intervention group) experienced the highest transition from “continue to drink” or “not sure” to “abstaining or stopping drinking”, increasing from 33% to 67%. In contrast, Kiamwangi showed the least transformation, moving from 48.4% to 51.6% (Figure 4). For the control group, Kabete and Muguga displayed the most significant negative transformation, with 100% of youth indicating they would continue drinking, and 58.3% and 48% remaining undecided, respectively.

Qualitatively, the findings from FGDs and KIIs indicated that youth lacked access to and awareness of screening services. Males and those with a secondary school education were at the highest risk of harm. Interviewees in the FGD and KII expressed a desire for structured health education to improve knowledge regarding the adverse effects. Additionally, youth in Gatundu indicated that structured health education enhanced their awareness and mitigated the adverse effects they faced.

## Discussion

Overall, the study noted significantly greater improvements in knowledge about alcohol-related harm among the intervention group compared to the control group. This was evidenced by a marked increase in the mean knowledge scores of the intervention group, rising from 28 to 36, yielding a difference-in-differences (DiD) value of 1.84, and a substantial effect size, as indicated by a Cohen´s d of 1.161. Additionally, the intervention group exhibited a notable behavioral shift, with the proportion of participants intending to abstain from alcohol increasing from 64.6% to 91.8%, and those intending to continue drinking decreasing from 4.5% to 0%. This improvement aligns with research suggesting that targeted educational interventions, including peer-to-peer education, effectively enhance knowledge about health risks [17].

Furthermore, the smaller improvement in knowledge levels in Kabete aligns with literature showing that general awareness may improve passively, but intentional, targeted, and active interventions are more effective in enhancing knowledge [26]. Additionally, the minimal changes in the control group correspond with findings that passive educational approaches have limited effects on behavioral outcomes [19]. The study findings are also supported by literature showing that health education helps prevent alcohol-related problems by addressing them proactively at an early stage [27].

**Limitations:** the study received frequent requests for monetary incentives from participants at the start. However, after raising awareness about the broader benefits of the study beyond financial incentives, their engagement improved.

**Interpretation:** from the findings, health education regarding alcohol effects should be community-led, well-structured to build the life skills of the youth, enhanced, and targeted to the most affected youth groups, particularly males. The findings align with literature indicating that interventions with detailed preventive information are effective in increasing knowledge and modifying behavior and attitude [17]. However, deliberate efforts to further address health and social consequences are known to improve knowledge retention [28]. This study's new findings demonstrate how to enhance and sustain young people's understanding of the consequences of alcohol, influencing behavior, and combating underage drinking. It also suggests a more effective structure for health education, particularly by incorporating life skills training to help youth resist the temptation of alcohol while utilizing local resources such as CHPs, recovered alcoholics, and youth peers in designing and implementing health education programs.

**Generalizability:** the study can be generalized to Kiambu County, but applying such an intervention to other regions depends on cultural, economic, and social differences. Future research should examine the use of technology in health education.

## Conclusion

The enhanced structured health education intervention helped address alcohol abuse in the intervention group by improving knowledge, promoting healthier behaviors, and reducing adverse effects. Targeted community-led interventions are needed for youth, especially those under 24 years, unemployed, and living without parents or guardians. The design and implementation should involve youth, community health workers, recovered alcoholics, government institutions, churches, schools, sports organizers, and chiefs.

### What is known about this topic

- Most studies have evaluated youth awareness of adverse effects using cross-sectional methods; furthermore, a few others rely on observational designs rather than intervention-based approaches;
- Passive educational methods, such as posters or one-time awareness campaigns, have a limited effect on behavior change, while interactive, community-driven programs have demonstrated a greater impact.

### What this study adds

- The study provides an evidence-based longitudinal intervention on how young people's understanding of the consequences of alcohol can be enhanced and sustained over time to influence behavior and combat underage drinking;
- The study also shows how better structuring of health education to incorporate life skills teaching, enhancement through regular monthly sessions, and the use of community resources, including CHPs and recovered alcoholics and youth in the design and implementation, can drive behavior change.
